# Resveratrol-loaded peptide-hydrogels inhibit scar formation in wound healing through suppressing inflammation

**DOI:** 10.1093/rb/rbz041

**Published:** 2019-10-30

**Authors:** Chen-Chen Zhao, Lian Zhu, Zheng Wu, Rui Yang, Na Xu, Liang Liang

**Affiliations:** 1 Institute of Biology and Medicine, College of Life Sciences and Health, Wuhan University of Science and Technology, Wuhan 430081, China; 2 The First College of Clinical Medical Science, China Three Gorges University & Yichang Central People’s Hospital, Yichang 443003 China

**Keywords:** resveratrol, peptide hydrogel, wound healing, inflammation

## Abstract

Scar formation seriously affects the repair of damaged skin especially in adults and the excessive inflammation has been considered as the reason. The self-assembled peptide-hydrogels are ideal biomaterials for skin wound healing due to their similar nanostructure to natural extracellular matrix, hydration environment and serving as drug delivery systems. In our study, resveratrol, a polyphenol compound with anti-inflammatory effect, is loaded into peptide-hydrogel (Fmoc-FFGGRGD) to form a wound dressing (Pep/RES). Resveratrol is slowly released from the hydrogel *in situ*, and the release amount is controlled by the loading amount. The *in vitro* cell experiments demonstrate that the Pep/RES has no cytotoxicity and can inhibit the production of pro-inflammatory cytokines of macrophages. The Pep/RES hydrogels are used as wound dressings in rat skin damage model. The results suggest that the Pep/RES dressing can accelerate wound healing rate, exhibit well-organized collagen deposition, reduce inflammation and eventually prevent scar formation. The Pep/RES hydrogels supply a potential product to develop new skin wound dressings for the therapy of skin damage.

## Introduction

Skin is the first protective barrier in the human body from the external environment. Rapid healing is a fundamental step to recover the barrier function when a wound occurs. However, skin wound healing often results in fibrotic scars and form keloids in adults [1, 2]. Wound healing is a complex process including three stages: inflammation, proliferation and remodelling [3]. There are growing evidences that macrophage-involved excessive inflammation is extensively related with the scar formation [4–6]. In the early stage of wound healing, macrophages express pro-inflammatory factors, including tumour necrosis factor-α (TNF-α), interleukins (IL)-6 and IL-1β, to engulf pathogens [7, 8]. But, in the following stages, macrophage subsets, such as CD206/CD301b macrophage and profibrotic macrophage, will appear and facilitate collagen deposition and excessive extracellular matrix components, resulting in fibrosis [9, 10]. Therefore, it is a possible strategy to reduce scar formation in wound healing through inhibiting inflammation.

Hydrogels possessed with several excellent properties has been widely used for biomedical applications [11–13]; their properties could be flexibly adjusted so as to meet different kinds of applications [14–17]. Peptide-based hydrogels have been proven to be ideal wound dressings due to their tunable mechanical stability, high water content, well biocompatibility and drug delivery capability. Peptides, composed of numbers of amino acids, can self-assemble to form porous three-dimensional (3D) fibrous scaffolds via ionic, hydrophobic and hydrogen bonding and π–π stacking [18–20]. In previous studies, peptide-hydrogels have been used to prevent postoperative scarring formation in glaucoma filtration surgery [21, 22]. Peptide-hydrogels also have exhibited accelerated healing of burn wounds [23, 24]. The well-organized and collagen-rich granulation tissue layers of peptide-hydrogels treatment results from the mimetic 3D structure to the extracellular matrix. On the other hand, the hydrogel serves as an excellent drug delivery matrix due to the porous mesh network, the hydration environment and gas permeability [25]. The scaffolds mimic extracellular matrix environments and provide a more natural environment for cells. Various peptides have been designed to establish 3D scaffolds for accelerating wound healing [23, 26, 27]. Moreover, the porous 3D microstructures supply an effective way for drug delivery [28]. In previous study, anti-proliferative 5-fluorouracil (5-FU) has been incorporated into peptide-hydrogel for inhibition of post-operative scarring formation in eyes [21].

Resveratrol is a natural polyphenolic compound. Many studies have demonstrated that resveratrol has physiological functions including anti-oxidant, anti-inflammatory, immunomodulatory and anti-cancer properties [29, 30]. Resveratrol has been shown to suppress fibroblast proliferation and induce apoptosis, which inhibits fibro genesis in keloids [31]. Resveratrol further reduced the production of pro-inflammatory factors and elevate the level of SIRT1 in severe burn [32]. Considering the pharmacological effects of resveratrol, we therefore hypothesize that resveratrol may inhibit excessive inflammation and accelerate skin wound healing.

**Scheme 1:**
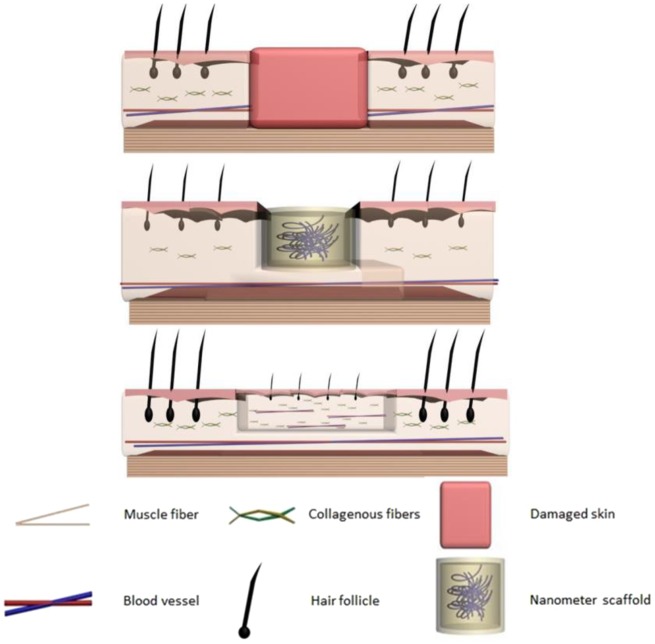
Schematic procedures of nanometer scaffold for the inhibition of post-operative scarring formation

In various wound dressing substrates, peptide-based hydrogels self-assembled with numbers of amino acids are particularly attractive because of their convenient assemble and 3D fibrous scaffolds with the similar microstructures as natural extracellular matrix [25]. It has also been proved to be an efficient drug delivery platform for wound dressing, anti-cancer and other therapeutics [21]. Herein, we designed and fabricated a resveratrol-loaded peptide-hydrogel as a skin wound dressings (Scheme 1). The *in vitro* inflammation response and *in vivo* scar formation were measured to evaluate the wound healing effect. Results demonstrated that our established skin would dressings could be effectively inhibit the formation of scar with high compatibility.

## Materials and methods

### Peptide synthesis and hydrogel preparation

The N-fluorenyl-9-methoxycarbonyl phenylalanine-phenylalanine-glycine-glycine-arginine-glycine-aspartic acid (Fmoc-FFGGRGD) short chain polypeptide powder (purity > 95%) was purchased from Bioyeargene Biotechnology Ltd (Wuhan, China). The peptide powders were dissolved in deionized water to obtain a stock solution. Series of different concentrations of peptide solutions (0.2, 0.5, 1, 1.5 and 2 wt%) diluted in deionized water were prepared and placed quiescently for 30 min at 37°C to explore the gelation concentration. The gelation state was observed by inverting tubes.

### Peptide-hydrogel characterization

The morphology of peptide-hydrogel (Pep) was characterized by field emission scanning electron microscopy (FE-SEM, FEI Nova 400 Nano) and high-resolution transmission electron microscopy (HR-TEM, JEM-2100, JEOL). For SEM characterization, the hydrogel was swelled with deionized water and lyophilized in a freeze dryer (SCIENTZ-10N). The samples were sprayed with gold before SEM observation. For TEM characterization, the hydrogel was evenly dispersed in ethanol and dripped onto the copper mesh. The observation was performed after natural air drying. The diameters of nanofibers in the hydrogels were measured by ImageJ software.

The oscillatory rheology experiment was performed on a rheometer (Physica RM301, Anton Paar). The hydrogel was placed in the centre of cone plate. The storage modulus (G') and loss modulus (G**″**) were recorded at the angular frequency range from 0.1 to 100 rad/s at 37°C.

### Resveratrol-loaded hydrogel preparation and release kinetics

Resveratrol (Sigma-Aldrich, USA) dissolved in DMSO (1 mg/ml) was added into peptide solution (2% wt) to obtain the resveratrol-loaded peptide-hydrogel. The samples with final resveratrol concentrations of 8 and 32 μg/ml in 2% wt peptide (Pep/8RES and Pep/32RES) were, respectively, prepared. Hydrogels were formed by placing samples at 37°C for 30 min.

The hydrogel samples were immersed into 1 ml phosphate saline buffer (PBS) to detect the release kinetic of resveratrol. The supernatants were collected after immersion for 1, 2, 3, 5, 7, 8, 10, 12 and 14 days, respectively. The resveratrol concentrations in the collected samples were detected by high-performance liquid chromatography (E2695, Waters).

### Cytotoxicity assay

Hydrogel extract was prepared according to ISO 10993-5. Briefly, the hydrogel was immersed into sterile water for sufficient swelling and then weighed. After removing sterile water, Dulbecco Modified Eagle Medium (DMEM, Gibco, USA) was added at the percentage of 0.1 g/ml (hydrogel/DMEM) and placed at 37°C for 48 h. NIH/3T3 and RAW 264.7 cells were inculcated into a 96-well plate (1.0 × 10^4^ cells/well). After cell adhesion, the hydrogel extract with 10% fetal bovine serum (Ginimi, USA) was added for cell culture. After culturing for 1, 2 and 3 days, cell viability was detected by CCK-8 kit (Beyotime, Shanghai) according to the instructions. The absorption value at 450 nm was determined by using a microplate reader (Spectra Maxi3, USA) to evaluate hydrogel cytotoxicity.

### Inflammation assay

The anti-inflammatory effect of resveratrol-loaded hydrogel was studied *in vitro* using lipopolysaccharide (LPS)-induced inflammation on RAW 264.7 macrophage cells. The peptide-hydrogels loaded with/without resveratrol were prepared on the bottom of a 6-well plate. Macrophage cells (1.0 × 10^6^ cells) were added into wells, and LPS was added after 6-h incubation. After 24-h or 48-h treatment, cells were collected for qRT-PCR assay to detect the mRNA expression of inflammatory cytokines TNF-α and IL-1β. The procedures are as follows. Total RNA was extracted by Trizol, and then the cDNA was obtained by using reverse transcription kit (PrimeScript RT reagent Kit, Takara, China). The analysis was performed on the CF96-Real-Time System (Bio-rad, USA) using SYBR Green Premix Ex Taq II (Takara, Beijing, China). The housekeeping gene GAPDH was employed as a control gene. The primers for the target genes are listed in Table 1.

**Table 1:** Primers sequences for qRT-PCR

Gene	Primer sequences (3′-5′)	Product size (bp)
GAPDH	F: GCAAGTTCAACGGCACAG	18
	R: GCCAGTAGACTCCACGACAT	20
TNF-α	F: CTCATTCCTGCTTGTGGC	18
	R: CACTTGGTGGTTTGCTACG	19
IL-1β	F: CTTCAGGCAGGCAGTATCA	19
	R: GTAGTGCAGTTGTCTAATGGG	21

After 24-h or 48-h treatment, the culture medium was collected to detect the concentration of TNF-α and IL-1β secreted by macrophages using ELISA kit (R&D Systems, USA). The absorbance at 450 nm was measured using a microplate reader, and the concentrations were obtained by measuring the standard curves.

### Rat model of skin wound healing

Adult male Sprague–Dawley rats (8 weeks old) were purchased from Centre for Animal Experiment in Wuhan University. The animal experiments were approved by Ethics Committee of Wuhan University of Science and Technology. The rats were anesthetized with 10% chloral hydrate at a dose of 0.3 g/100 g. A round full-thickness skin wound with a diameter of 2.0 cm was prepared on the dorsal surface after shaved. The rats were randomly divided into four groups with more than six rats in each group. Three groups were dressed with 200 μl Pep, Pep/8RES and Pep/32RES, respectively. One group without any treatment was used as negative control (CTL). All rats were housed individually and monitored daily for eating and drinking. To evaluate the rate of wound healing, the wound region was captured by a digital camera each day from Day 7 to Day 14. The photographs were analysed using Image J software.

### Histological examination

After 7 days, the rats were euthanized and the skin tissues were collected from the wound regions. Normal skin tissue (NC) was collected as a control. The tissues were washed with PBS, fixed with 4% polyformaldehyde, dehydrated with gradient ethanol and embedded in paraffin wax. The paraffin slices were prepared by a slicing machine and then stained in haematoxylin and eosin (HE) or Masson Trichrome (Google Biotech., Wuhan, China). The images were captured with a microscopy (Pannoramic MIDI, 3D HISTWCH). The skin thickness was measured from the images by Image J software.

Angiogenesis was evaluated by immunohistochemically stained with CD31 PECAM-1 polyclonal antibody (R&D Systems, USA). Paraffin slices were dewaxed in xylene and rehydrated in gradient ethanol. The sections were washed with distilled water and then immersed in 0.01 mol/l sodium citrate buffer (pH 6.0) for epitope repair. After immunohistochemically stained, the sections were visualized with 3,3′-diaminobenzidine (DAB) colour reagent and slightly re-stained with H&E. The slices were dehydrated and sealed with neutral resin for the imaging. The average optical density of CD31 was measured by Image J software to evaluate the expression level of CD31.

### Statistical analysis

All the experiments were conducted in triplicate, and the data were expressed as mean ± SD. To determine the statistical significance of differences between the study groups, the two sample *t*-test was applied. The statistical significance was defined as *P* < 0.05.

## Results

### Peptide gelation and characterization

**Figure 1:**
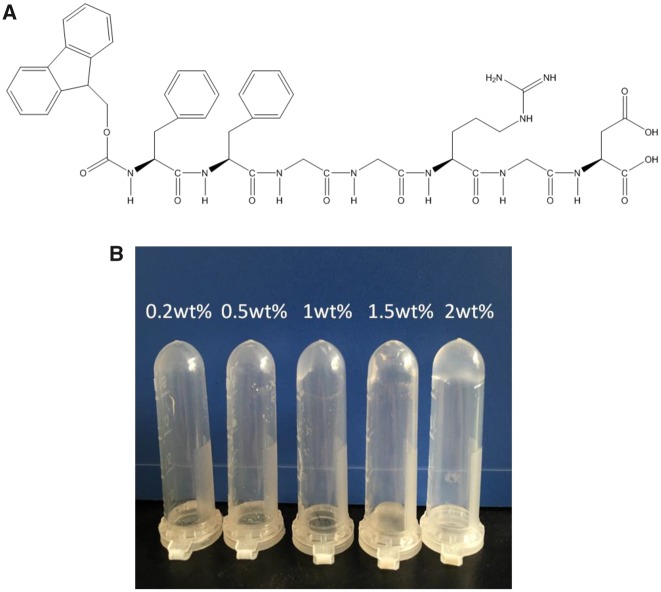
(**A**) Molecular structure of Fmoc-FFGGRGD. (**B**) Gelation of peptide solutions with the concentrations of 0.25, 0.5, 1, 1.5 and 2% wt, respectively (30°C, 30 min)

The molecular structure of the peptide Fmoc-FFGGRGD is shown in Fig. 1A. The peptide gelation results depicted in the inverted tubes (Fig. 1B) showed that peptides-hydrogels were formed when the concentration of peptide is higher than 1% wt after peptide solutions stayed for 30 min at 37°C.

**Figure 2:**
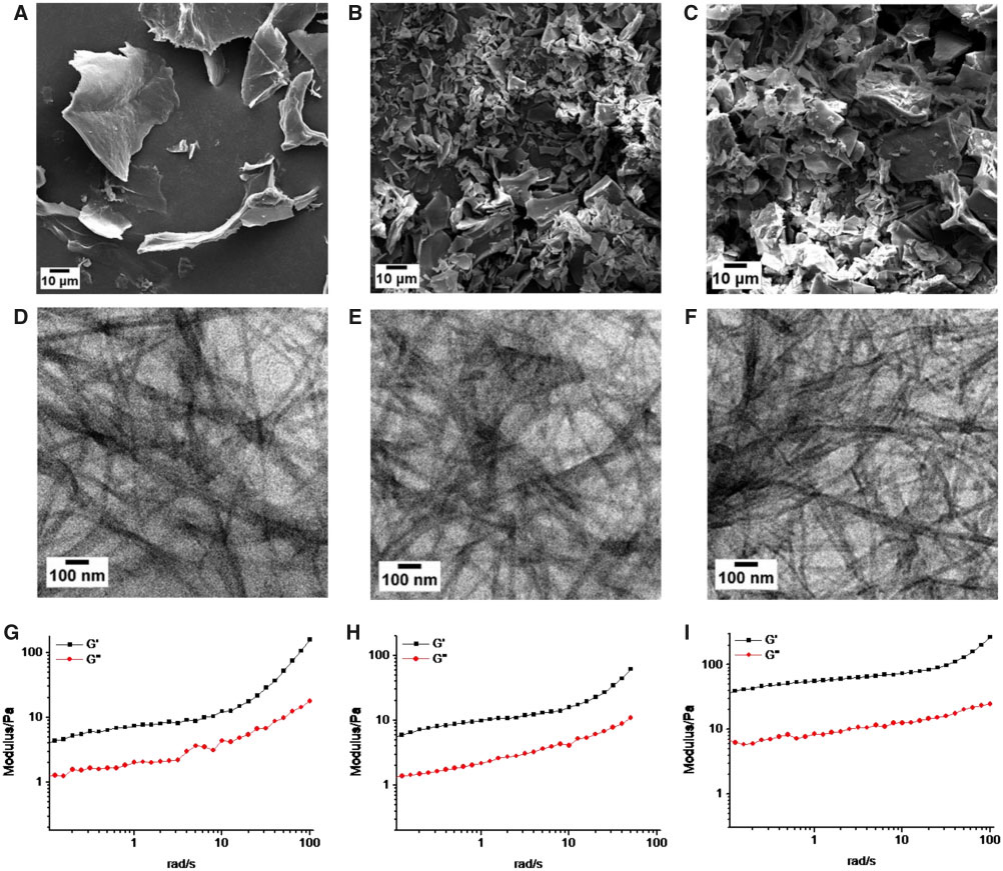
Morphology characterization and oscillatory rheology analysis of peptide-hydrogels. (**A**–**C**) SEM images, respectively, for 1% wt, 1.5% wt and 2% wt hydrogels from left to the right. (**D**–**F**) HR-TEM images, respectively, for 1 wt%, 1.5% wt, and 2% wt hydrogels from left to the right. (**G**–**I**) The curves of storage modulus (G′) and loss modulus (G′) of hydrogels with concentration of 1% wt (G), 1.5% wt (H) and 2% wt (I) from left to the right

The microstructures of the peptide-hydrogels (1, 1.5 and 2% wt) were characterized by SEM and TEM (Fig. 2). The SEM images (Fig. 2A–C) showed that the freeze-dried hydrogels existed in the form of sheet. The TEM images (Fig. 2D–F) showed the porous and nanofibrous structures in the internal hydrogels. The density of peptide nanofibers got thicker with the increase of peptide concentration. Interestingly, the nanofiber diameters of the three samples were 25.52 ± 6.38, 26.12 ± 6.11 and 26.49 ± 7.46 nm, respectively, showing no significant difference. The oscillatory rheology results (Fig. 2G–I) showed that the storage modulus (G′) is higher than loss modulus (G″) for the three samples, suggesting a gel state of peptide-hydrogels. As the peptide concentration increases, the storage modulus get higher, indicating more stable gel state. Therefore, in the following studies, we chose 2% wt peptide-hydrogels as wound dressing substrate.

### Resveratrol-loaded peptide-hydrogels and drug release

**Figure 3:**
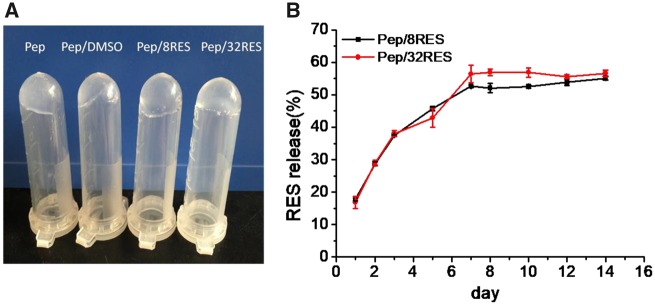
Resveratrol-loaded peptide-hydrogels gelation (**A**) and resveratrol release kinetics (**B**)

Resveratrol was incorporated into peptide-hydrogels in the gelation process when adding resveratrol into 2 wt% peptide solutions. The resveratrol-loaded hydrogels containing 8 μg/ml (Pep/8RES) and 32 μg/ml (Pep/32RES) resveratrol kept the hydrogel state well (Fig. 3A). When the samples were immersed into PBS solution, the resveratrol was released slowly and the release proportion was accumulated with time (Fig. 3B). The highest release proportions of Pep/8RES and Pep/32RES were (55.8 ± 1.5%) and (56.6 ± 1.0%), respectively, showing no significant difference. In the Day 14, the release proportions of Pep/8RES and Pep/32RES at were (55.1 ± 0.6%) and (56.6 ± 1.0%), respectively.

### Cytotoxicity assessment

**Figure 4:**
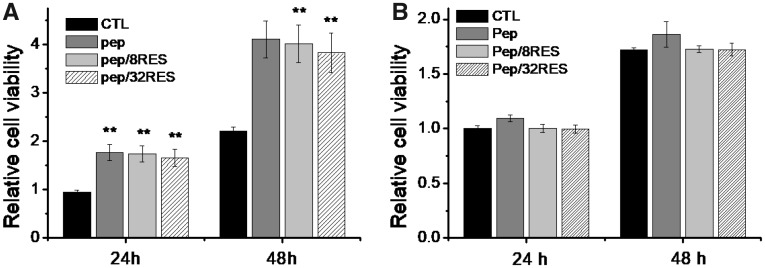
Cytotoxicity of resveratrol-loaded peptide-hydrogels to NIH/3T3 (**A**) and RAW 264.7 cells (**B**)

In order to assess the biosafety of resveratrol-loaded peptide-hydrogels, we evaluated the cytotoxicity of hydrogel extract liquids to NIH/3T3 and RAW 264.7 cells *in vitro* by CCK-8 method. The results (Fig. 4) showed that the cell proliferation of NIH/3T3 was significantly prompted for Pep, Pep/8RES and Pep/32RES compared to control. But cell proliferation of RAW 264.7 cells showed no significant difference between all samples. It suggested that resveratrol-loaded peptide-hydrogels in our studies are safe as wound dressing.

### Anti-inflammation effect

**Figure 5:**
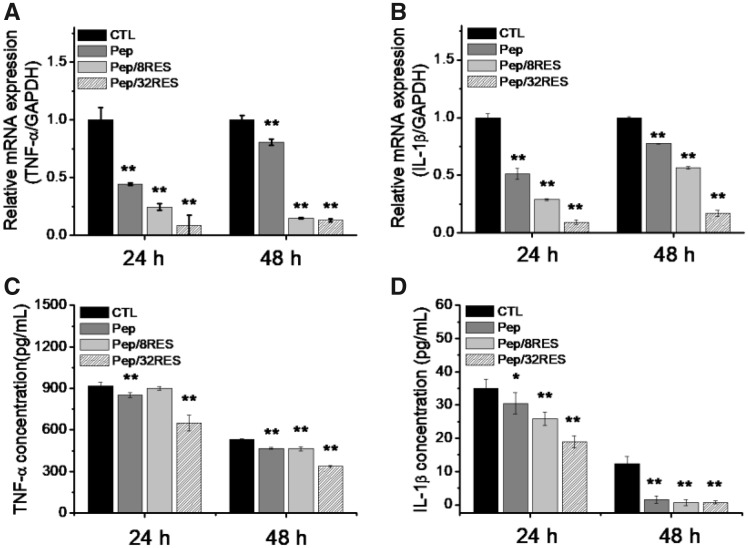
Anti-Inflammatory effect of peptide-hydrogels characterized by mRNA gene expression levels of TNF-α (**A**) and IL-1β (**B**) detected by RT-PCR and the production of inflammatory cytokines TNF-α (**C**) and IL-1β (**D**) detected by ELISA

Macrophages are the main inflammatory cells involved in wound healing process. In order to reveal the influence of resveratrol-loaded peptide-hydrogel on inflammation effect, the gene expression and production of inflammatory factors by LPS-induced macrophages, including TNF-α and IL-1β, were detected by RT-PCR and ELISA after 24-h and 48-h treatment. As shown in Fig. 5A and B, the gene expressions of TNF-α and IL-1β were all down-regulated for Pep and Pep/RES compared to CTL and reduced even more for Pep/RES samples. Simultaneously, the reduction ratio increased with the amount of resveratrol.

The ELISA results (Fig. 5C and D) showed the same tendency as RT-PCR results. It suggested that peptide and resveratrol can both inhibit the production of inflammatory factors. Moreover, the ELISA results showed that the production of TNF-α and IL-1β decreases with treatment time, indicating the sustaining inhibitory effect of resveratrol-loaded hydrogels on inflammation.

### Wound healing

**Figure 6:**
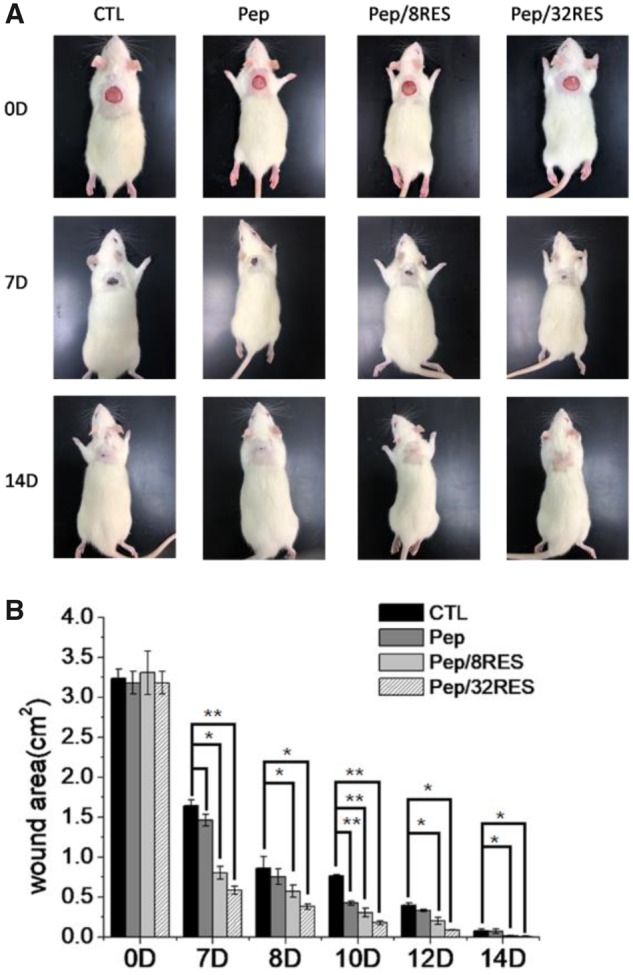
Wound healing monitoring with peptide-hydrogel dressing treatment. (**A**) Photographic monitoring of wound contraction in rat skin wound model after dressing treatment for 7 and 14 days. (**B**) Wound area measurement and analysis

In order to elucidate the effect of peptide-hydrogels on skin wound healing, a skin wound model on a rat with a surface area of approximately 3.14 cm^2^ was fabricated. And then, the wound closure was observed after painting peptide-hydrogels as wound dressings. Figure 6 showed the photographic monitoring and area measurement of wound contraction. By Day 7, wound areas for CTL, Pep, Pep/8RES, and Pep/32RES were, respectively, decreased to 1.64 ± 0.07, 1.46 ± 0.07, 0.80 ± 0.08 and 0.58 ± 0.05 cm^2^, showing significant differences. 90% wound closure was obtained at Day 8 for Pep/32RES, whereas CTL and Pep were demonstrated at Day 12.

**Figure 7:**
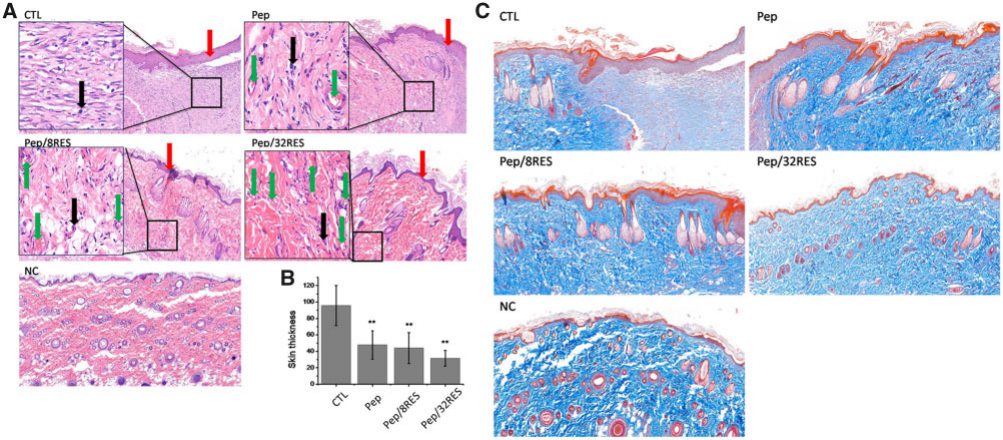
Histological analysis of wound surface after treatment for 7 days. (**A**) H&E staining images. The red arrows mean newly formed skin surface. The black arrows mean infiltration of inflammatory cells. The green arrows mean neovascularization. (**B**) The thickness of newly formed skin surface. (**C**) Masson trichrome staining images

Rats were sacrificed after Day 7 for histological analysis by H&E and Masson’s trichrome staining to assess the newly formed skin tissue. In Fig. 7A, wound treated without dressing (CTL) resulted in the thickest granulation tissue in the wound area. In contrast, wounds treated with Pep or Pep/RES dressing resulted in a thinner granulation tissue (Fig. 7B). Severe inflammatory cell infiltration and edema were observed in the CTL group, whereas slight infiltration of inflammatory cells, neovascularization and re-epithelialization was observed in the peptide-hydrogel treatment groups.

Moreover, the resveratrol-loaded hydrogel treatment group showed better connective tissue reorganization, and the disordered dense structure began to become orderly, especially for Pep/32RES. Masson Trichrome staining results (Fig. 7C) showed that the collagen arrangement in the CTL group was dense and disordered, showing the formation of scars. In contrast, the collagen deposition of the regenerated skin treated with peptide-hydrogel was similar to that of the normal skin, showing an orderly arrangement. The collagen deposition in Pep/8RES and Pep/RES groups were more close to the normal skin (NC). Histological staining of regenerated skin showed that resveratrol-loaded peptide-hydrogels provided a more favourable environment for inhibiting scar formation and accelerating wound healing.

**Figure 8:**
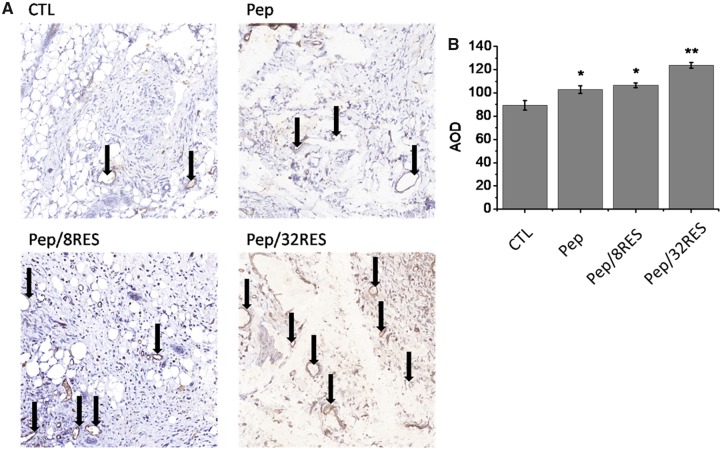
Analysis of angiogenesis. (**A**) CD31 immunohistochemical staining of wound surface after dressing treatment for 7 days. (**B**) The average optical density (AOD) of CD31 measured by Image J software

The immunohistochemically staining of CD31, a marker of vascular endothelial differentiation, was performed to evaluate the angiogenesis level (Fig. 8A). The results showed that there were only a few newly formed blood vessels in the CTL group and some tiny vessels were observed in Pep group. But Pep/8RES and Pep/RES groups showed a large number of blood vessels and increased blood vessel size (Fig. 8B), which is consistent with the results of Masson trichrome staining.

## Discussion

Scar formation impedes the skin wound healing and results in keloid in adults. Many studies have demonstrated that the excessive inflammation is related with scar formation, especially macrophage-involved inflammation [33]. Therefore, inhibiting excessive inflammation and reducing scar formation is an effective way for prompting wound healing. Resveratrol is widely explored due to its effects of anti-oxidant, anti-inflammatory and anti-cancer [34–36]. Therefore, in our studies, we designed and fabricated the resveratrol-loaded peptide-hydrogels and evaluated the effect on wound healing and scar formation.

The Fmoc-FFGGRGD peptide we designed is self-assembled when the concentration is higher than 1% wt at 37°C (Fig. 1B). The inner peptide fiber density (Fig. 2D–F) and the gel stability (Fig. 2G–I) are increased with the concentration. But the diameters of peptide fibrils for different concentration showed no significant difference. As explored in previous studies, the driving forces that favour intermolecular binding for peptide gelation are weak interactions including hydrophobic effects, ionic interactions, hydrogen binding, π–π stacking, cation–π interactions and intrinsic sequence propensity [25, 29, 37]. Considering the chemical structure of the peptide (Fig. 1A), at least three interactions exist in our hydrogel. The main driving force for self-assembling of peptide hydrogelators is hydrophobic effect. It favours the formation of primary micelles [38]. The FF motif contributes to π–π stacking and amino and carboxyl groups in aspartic acid contribute to hydrogen bonding. They provide directionality in the self-assemble process of nanofibrils. Therefore, the property of nanofibrilsis dependent on the structure of peptide sequence, which explains the TEM results in Fig. 2. But the strength of hydrogel is sensitive to environmental conditions, such as temperature and concentration [39]. With the peptide concentration increased, the interactions between molecules are enhanced due to the increase of molecule number. Two percent wt peptide-hydrogels showed the highest elastic modulus and most stable gel state in our studies.

Resveratrol has been determined as a potential anti-inflammatory agent. Therefore, we added resveratrol into hydrogels to realize *in situ* release. After adding 8 or 32 μg/ml resveratrol into 2% wt peptide solution, the gelation is still formed and hydrogels are stable at 37°C (Fig. 3A). The release kinetics showed that resveratrol is slowly released when hydrogels are immersed into PBS and the release rate at Day 7 is about 50% for both Pep/8RES and Pep/32RES samples (Fig. 3B). Although the loaded resveratrol amounts of the two samples are different, the release ratios showed no significant difference. The release kinetic can be explained by the synergistic effect of network mesh size, surface area to volume ratio, the amount of drug and the interaction between drug and the hydrogel [40]. In our study, only the loaded resveratrol amounts for Pep/8RES and Pep/32RES samples are different, so the release amount is mainly controlled by the loaded amount. This result suggested that resveratrol can be released *in situ* and is a promising agent as wound dressing.

Cytotoxicity is a necessary evaluation indicator for wound dressing products. The cell proliferation results (Fig. 4) demonstrated that the Pep/RES samples prompted fibroblastic NIH/3T3 cell proliferation while had no obvious influence for macrophage RAW 264.7 cells when treated with hydrogel extract liquids for 48 h. But the inflammatory factors secreted by LPS-induced macrophages are significantly inhibited by Pep/RES samples, both in gene expression level and production level (Fig. 5). This indicated that Pep/RES samples play important roles in inhibiting inflammation effect *in vitro*. To further explore the wound healing *in vivo*, rat skin wound model was established for histological analysis. For different wound dressing treatment, the skin wound healing exhibited significant difference in healing rate and Pep/32RES showed the highest wound contraction rate with 90% wound closure at day 8 (Fig. 6). The histological analysis indicated that Pep/RES dressing demonstrated less infiltration of inflammatory cells and better re-epithelialization (Fig. 7). The collagen deposition in Pep/RES samples was much more regular and thinner, indicating a more natural extracellular matrix formation. Regular collagen deposition is an indicator of inhibiting scar formation. More importantly, Pep/RES samples showed more newly formed blood vessels (Fig. 8). Form the above, it is concluded that Pep/RES wound dressing has effective influence in prompting skin wound healing via inhibiting inflammation.

The phenomenon can be explained by the synergistic function of resveratrol and peptide. Peptide-hydrogels are biocompatible materials due to their natural amino acid component [41]. As the immune system plays important roles in skin wound repair and resveratrol is an anti-inflammatory agent, the inflammatory effect in skin wound healing is obviously inhibited by the *in* *situ* released resveratrol. The mechanism has been explored in various studies and the NAD^+^-dependent protein deacetylasesirtuin 1 (SIRT1) has been demonstrated to be involved in the deposition of extracellular matrix proteins resulting in hypertrophic scars [42]. Resveratrol has been determined to activate SIRT1, but the activation *in vivo* may be not direct [43, 44]. Therefore, owe to the package and slow release function of peptide-hydrogel, resveratrol may reduce scar formation and accelerate skin wound healing by activating SIRT 1 to reduce excessive collagen formation.

## Conclusions

It is an important issue for skin wound healing to inhibit scar formation and excessive inflammation. In our report, we designed and fabricated a resveratrol- loaded peptide-hydrogel (Pep/RES) as skin wound dressing. The *in situ* released resveratrol from the hydrogel provides an anti-inflammatory effect *in vitro* and *in vivo*. The Pep/RES dressing exhibited accelerated wound healing rate, less inflammation, well-organized collagen deposition and eventually reduced scar formation. The Pep/RES hydrogels supply a potential product for new skin wound dressings. 
